# Genome-Wide Identification of Rapid Alkalinization Factor Family in *Brassica napus* and Functional Analysis of BnRALF10 in Immunity to *Sclerotinia sclerotiorum*

**DOI:** 10.3389/fpls.2022.877404

**Published:** 2022-05-03

**Authors:** Yu-Han He, Zhuo-Ran Zhang, You-Ping Xu, Song-Yu Chen, Xin-Zhong Cai

**Affiliations:** ^1^Key Laboratory of Biology of Crop Pathogens and Insects of Zhejiang Province, Institute of Biotechnology, College of Agriculture and Biotechnology, Zhejiang University, Hangzhou, China; ^2^Centre of Analysis and Measurement, Zhejiang University, Hangzhou, China; ^3^Hainan Institute, Zhejiang University, Sanya, China

**Keywords:** *Brassica napus*, FER, plant immunity, proteome, RALF, *Sclerotinia sclerotiorum*, signaling

## Abstract

Rapid alkalinization factors (RALFs) were recently reported to be important players in plant immunity. Nevertheless, the signaling underlying RALF-triggered immunity in crop species against necrotrophic pathogens remains largely unknown. In this study, RALF family in the important oil crop oilseed rape (*Brassica napus*) was identified and functions of BnRALF10 in immunity against the devastating necrotrophic pathogen *Sclerotinia sclerotiorum* as well as the signaling underlying this immunity were revealed. The oilseed rape genome carried 61 RALFs, half of them were atypical, containing a less conserved YISY motif and lacking a RRXL motif or a pair of cysteines. Family-wide gene expression analyses demonstrated that patterns of expression in response to *S. sclerotiorum* infection and DAMP and PAMP treatments were generally RALF- and stimulus-specific. Most significantly responsive *BnRALF* genes were expressionally up-regulated by *S. sclerotiorum*, while in contrast, more *BnRALF* genes were down-regulated by BnPep5 and SsNLP1. These results indicate that members of BnRALF family are likely differentially involved in plant immunity. Functional analyses revealed that BnRALF10 provoked diverse immune responses in oilseed rape and stimulated resistance to *S. sclerotiorum*. These data support BnRALF10 to function as a DAMP to play a positive role in plant immunity. BnRALF10 interacted with BnFER. Silencing of *BnFER* decreased BnRALF10-induced reactive oxygen species (ROS) production and compromised rape resistance to *S. sclerotiorum*. These results back BnFER to be a receptor of BnRALF10. Furthermore, quantitative proteomic analysis identified dozens of BnRALF10-elicited defense (RED) proteins, which respond to BnRALF10 in protein abundance and play a role in defense. Our results revealed that BnRALF10 modulated the abundance of RED proteins to fine tune plant immunity. Collectively, our results provided some insights into the functions of oilseed rape RALFs and the signaling underlying BnRALF-triggered immunity.

## Introduction

As sessile organisms, plants have evolved sophisticated communication systems to deal with constantly changing environmental conditions. Under this scenario, small secreted peptides serve as signal molecules to orchestrate a plethora of plant processes such as development and immune responses (Olsson et al., 2019). Rapid alkalinization factors (RALFs) are small cysteine-rich secreted peptides that are involved in multiple physiological and developmental processes, ranging from cell elongation to modulation of immune responses, and thus are also referred to as plant peptide hormones (Blackburn et al., 2020). RALFs were first discovered over 20 years ago due to their ability to cause rapid alkalinization of the extracellular compartment of tobacco cells (Pearce et al., 2001). Since then, RALFs were found to be ubiquitous in plants and microbes. Genome-wide analyses have identified 37 RALFs in *Arabidopsis* (Abarca et al., 2021), 765 in 51 plant species (Campbell and Turner, 2017), 124 in 7 Rosaceae species (Zhang H. et al., 2020), 17 in *Fusarium* species and 18 in 6 nematode species (Thynne et al., 2017; Zhang X. et al., 2020). The wide distribution of RALFs highlighted their functional importance. RALFs are well known pivotal contributors to plant growth and development (Haruta et al., 2014; Du et al., 2016; Dressano et al., 2017; Abarca et al., 2021; Zhu et al., 2021). However, relatively, role and mechanisms of RALFs in plant immunity are much less studied. Recently, two studies in Arabidopsis revealed that RALFs affect immune responses such as reactive oxygen species (ROS) generation and pathogen associated molecular pattern (PAMP)-triggered immunity (PTI) (Stegmann et al., 2017; Abarca et al., 2021). RALFs are recognized by its receptor FERONIA (FER) (Haruta et al., 2014). They form a complex together with LLGs (Xiao et al., 2019), and negatively modulate flg22-triggered immunity to *Pseudomonas syringae* (Stegmann et al., 2017). In contrast to the recognition of RALFs, the signaling downstream the recognition is still largely unclear.

Typically, RALF proteins are small molecules with an average full length of 80–120 amino acids. They execute their functions in their mature form released via cleaving by proteases (Matos et al., 2008; Srivastava et al., 2009). *Arabidopsis* RALF22 and RALF23 are cleaved at a di-basic RRXL site by site-1 protease (AtS1P), a plant subtilisin-like serine protease. This processing contributes to the function of these two RALFs on salt tolerance and plant immunity regulation, respectively (Stegmann et al., 2017; Zhao et al., 2018). Fourteen out of 37 *Arabidopsis* RALFs display the predicted S1P cleavage site, suggesting the conservation of this processing mechanism in the RALF family (Abarca et al., 2021). In addition to the RRXL cleavage site, the mature RALFs also contain other conserved motifs, such as the YISY motifs near the N-terminus and the four cysteine residues at the C-terminus (Campbell and Turner, 2017). The YISY motif of RALFs is crucial for their biological functions and receptor binding. Replacing the isoleucine with alanine significantly compromised their ability of alkalization and the root growth inhibition (Pearce et al., 2010; Xiao et al., 2019). The four conserved cysteines form two intramolecular disulfide bonds, which play a key role in maintaining the three-dimensional conformation and biological activity of RALFs. Mutation of the first two cysteine residues of *Arabidopsis* RALF1 influenced its root growth and PTI inhibition activity (Zhang X. et al., 2020), while replacement of the four cysteines of RALF4 with alanine weakened its binding to LRR extensin proteins, thereby affects pollen tube germination (Moussu et al., 2020). Nevertheless, it is noticeable that atypical RALFs that do not carry canonical RRXL, YISY and/or four conserved cysteines exist widely in plants (Campbell and Turner, 2017). Functions and mechanisms of these atypical RALFs remain largely unknown.

*Sclerotinia sclerotiorum* (Lib.) de Bary is a devastating soil-borne necrotrophic fungal pathogen. It causes diseases on a broad range of hosts including economically important crops such as oilseed rape (*Brassica napus*), sunflower and soybean, resulting in substantial yield losses (Ding et al., 2021). Oilseed rape is one of the most important oil crops globally. White mold disease caused by *S. sclerotiorum* is the most severe disease in oilseed rape. Nevertheless, the mechanisms underlying immunity to *S. sclerotiorum* in oilseed rape remain largely unexplored. In this study, we identified the *RALF* gene family in oilseed rape, and defined the expression patterns of the whole BnRALF family in response to *S. sclerotiorum* infection and PAMP/DAMP treatments. Furthermore, we conducted a series of analyses to reveal the functions and mechanisms of BnRALF10, one of the BnRALFs which highly responded to *S. sclerotiorum* in oilseed rape. Our results demonstrated that BnRALF10 acted as a DAMP molecule to elicit multiple typical immune responses and triggered immunity against *S. sclerotiorum*. It modulated accumulation level of various defense regulators to stimulate immunity against *S. sclerotiorum*. This RALF exhibited potential for breeding for improved disease resistance against necrotrophic pathogens.

## Materials and Methods

### Identification of Putative Rapid Alkalinization Factor Proteins in *Brassica napus*

Protein sequences of *Arabidopsis* RALFs were downloaded from TAIR database^1^. *Arabidopsis* RALF protein sequences were used as queries to search their orthologs in *B. napus* genomes using BLASTp program in NCBI database^2^ with default settings. All retrieved protein sequences were examined for the presence of conserved motifs and redundant sequences were removed. The physico-chemical properties of BnRALF proteins were predicted using ExPASy Compute pI/Mw tool^3^ (Bjellqvist et al., 1993).

### Construction of Rapid Alkalinization Factor Alignments and Phylogenetic Trees

Multiple sequence alignment of the full-length RALF protein sequences were created using the MUSCLE algorithm with the MEGA X software (Kumar et al., 2018). Phylogenetic tree was constructed with the MEGA X software by using the unweighted pair-group method with arithmetic means (UPGMA) algorithm following the Jones-Taylor-Thornton (JTT) model. Bootstrapping was performed 1,000 times. All other parameters were left as default. The inferred tree were visualized using iTOL^4^ (Letunic and Bork, 2019). The multiple mature peptides alignment was displayed using the GeneDoc2.7.0 and the results were manually edited^5^. WebLogo3^6^ was used to provide a visual summary of conserved residues within the alignments.

### Plant Materials and Pathogen Inoculation Analysis

*Brassica napus* plants were grown in growth cabinets at 23°C under a 14 h/10 h light/dark photoperiod. Fresh sclerotia of *S. sclerotiorum* strain UF1 (Xu T. T. et al., 2018) were cultured at 23°C on potato dextrose agar medium (PDA) to produce mycelia, which were transferred to new PDA plates and grown for 2 days. The PDA plugs containing young *S. sclerotiorum* mycelia were punched to inoculate the plant leaves. Area of disease lesions was measured using the ImageJ image analysis software^7^. For disease resistance evaluation, at least five plants for each genotype were examined and the experiments were conducted three times independently.

### Peptides

BnRALF10 (AQKYVSYGAMRKNSVPCSRGASYNCQRGAQNP YRGCSTIRCR), BnPep5 (SLNVSSKLTRKLPVSSGKRGGVN) and *S. sclerotiorum* necrosis and ethylene-inducing peptide 1 (SsNep1)-like peptide 1 (SsNLP1) (GIMYAWYFPKDQPAAGN VVGGHRHDWE) were synthesized by Chinapeptides, Suzhou, China.

### RNA Isolation and Gene Expression Analysis

For gene expression analysis, the oilseed rape leaves were sampled at 7 h post inoculation with *S. sclerotiorum* and 4 h post infiltration with 1 μM SsNLP1 and 200 nM BnPep5. Total RNA was extracted using Trizol reagent (Vazyme, Nanjing, China) following the manufacturer’s procedure. Quantitative real-time PCR (qRT-PCR) was performed using the StepOne Real-Time PCR system (Applied Biosystems, United States) with SYBR Green PCR Master Mix (TaKaRa, Dalian, China). The relative fold changes were calculated using the 2^–ΔΔCt^ method as previously described (Cao et al., 2016), with two technical replicates for each of the three biological replicates. The housekeeping gene *BnActin7* was used as an internal control. The heatmaps were created by TBtools (Chen et al., 2020) on the basis of the log_2_-fold-transformed data. Primers used for qRT-PCR are listed in Supplementary Table 1.

### Turnip Yellow Mosaic Virus-Induced Gene Silencing Manipulation Procedure

Turnip yellow mosaic virus-based VIGS experiment was performed as previously described with some modifications (Pflieger et al., 2008). The TYMV-derived construct pTY-S (controlled by 35S promoter) contained the full length cDNA of TYMV, of which a unique *Sna*BI restriction site was inserted into the coat protein. Both sense and antisense versions of a specific 40 bp fragment from the coding region of *BnRALF10* were designed to form self-hybridized palindromic oligonucleotide according to the reported method (Pflieger et al., 2008). The pTY-*BnRALF10* constructs were obtained by inserting the self-hybridized palindromic oligonucleotide of *BnRALF10* into the *Sna*BI site of pTY-S. The synthesis of the self-hybridized palindromic oligonucleotide and construction of pTY-*BnRALF10* recombinant vector were completed by the company (Tsingke, Hangzhou, China). pTY-*BnPDS* (*B. napus* phytoene desaturase) vector was constructed to monitor the silencing efficiency. The sequence information of the oligonucleotides was listed in Supplementary Table 1. Two-week-old *B. napus* cultivar Zhongshuang 11 (ZS11) seedlings were used for VIGS experiments with pTY, pTY-*BnPDS* and pTY-*BnRALF10* plasmid (6 μg). Plants were placed in the dark 24 h prior to inoculation, and were mechanically inoculated by rubbing the upper surface of three rosette leaves with 8 μL of inoculum, using a gloved finger and celite (Sigma-Aldrich, St. Louis, MO, United States). The inoculated *B. napus* seedlings were then transferred into the chamber for silencing. Plants were checked for *BnRALF10* gene silencing by qRT-PCR, and meanwhile the same plants were used for the pathogen inoculation analysis.

### Reactive Oxygen Species Assay

Leaf disks (3 mm in diameter) of *B. napus* were collected and dipped in 96-well plates containing sterile water in the dark overnight. The next day, the water was replaced by a solution containing 11 μM L-012 (FUJIFILM Wako Pure Chemical, Osaka, Japan), 20 μg/mL horseradish peroxidase (HRP, Sigma-Aldrich) and 1 μM BnRALF10 peptide. Luminescence was measured for the indicated time period using a Microplate Luminometer (Titertek Berthold, Bad Wildbad, Germany). ROS production is either displayed as the integration of total photon counts or as the progression of photon counts.

### Measurement of Cytosolic Calcium

*Arabidopsis thaliana* plants expressing cytosolic apoaequorin used for [Ca^2+^]_cyt_ measurements were kindly provided by Prof. Yan Liang (Zhejiang university, China). Seventy microliters of ddH_2_O containing 10 mM coelenterazine h (Sigma-Aldrich, St. Louis, MO, United States) was added per well in a 96-well plate. Vertically grown 5-day-old transgenic seedlings were individually transferred to each well and incubated overnight in the dark at room temperature. At the next day, 30 μL of ddH_2_O containing BnRALF10 peptide was added to each well, the final concentration of BnRALF10 was 1 mM, and the chemiluminescent signal was immediately recorded using a Microplate Luminometer (Titertek Berthold, Bad Wildbad, Germany). Photon counts were converted to calcium concentration.

### Mitogen-Activated Protein Kinases Phosphorylation Assay

Seven-day-old *Arabidopsis* Col-0 seedlings were immersed in liquid 1/2MS medium overnight. BnRALF10 peptide was then added to a final concentration of 1 μM for 5–30 min induction. After induction, the seedlings were snap-frozen in liquid nitrogen and ground to a fine powder, from which total protein was extracted by suspension in the extraction buffer containing 20 mM HEPES (pH 7.5), 150 mM NaCl, 5 mM MgCl_2_, 0.5% Triton X-100, and 1 × protease inhibitor cocktail (HuaBio, Hangzhou, China). An anti-phospho p44/p42 mitogen-activated protein kinases (MAPK) antibody (Cell Signaling Technology, Danvers, MA, United States) was used to detect active MPK6 and MPK3 via immunoblotting.

### Yeast Two-Hybrid Assay

The extracellular domain of BnFER (XP_013734398.1) and the full-length BnRALF10 were cloned into the bait vector pGBKT7 and the prey vector pGADT7, respectively. The resulting plasmids BD-BnFER and AD-BnRALF10 were co-transformed into the yeast strain Y2H Gold, while pGBKT7 and AD-BnRALF10 were co-transformed into the same strain to serve as a negative control. Presence of the transgenes was confirmed by growth on an SD-Leu-Trp agar plate. To assess protein interactions, the transformed yeast cells were suspended in liquid SD-Leu-Trp medium to an optical density at 600 nm of 1. Five microliters of suspended yeast cells was dropped onto an SD-Ade-His-Leu-Trp agar plate. The resulting agar plate was incubated at 30°C and observed for yeast growth. The primers used for the yeast two-hybrid (Y2H) assay are listed in Supplementary Table 1.

### Bimolecular Fluorescence Complementation Assay

For the bimolecular fluorescence complementation (BiFC) assay, the coding sequences of BnRALF10 and BnFER were cloned into the vectors cYFP and nYFP, respectively. *Agrobacterium* suspensions carrying each plasmid were mixed at 1:1 ratio and were then infiltrated into *Nicotiana benthamiana* leaves. Confocal microscopy images were captured at 2 days after infiltration. The primers used for the BiFC are listed in Supplementary Table 1.

### Luciferase Complementation Imaging Assay

A luciferase complementation imaging (LCI) assay was conducted. The full-length BnFER and BnRALF10 were cloned into the vectors NLuc and CLuc, respectively. Paired constructs of BnRALF10-CLuc and BnFER-NLuc were transiently co-expressed in the leaves of *N. benthamiana* through *Agrobacterium*-mediated co-infiltration. The primers used for this assay are listed in Supplementary Table 1.

### Protein Extraction

Total proteins from each sample were extracted by the modified method of trichloroacetic acid (TCA)-acetone precipitation (Cao et al., 2016). Approximately 1 g samples were pulverized using a mortar and pestle in liquid nitrogen, and the fine powder was suspended overnight at −20°C in 30 ml of ice-cold acetone containing 10% (w/v) TCA and 0.07% (w/v) DL-Dithiothreitol (DTT). The protein precipitate was pelleted by centrifugation at 35,000 × *g* for 1 h and resuspended in 30 ml of ice-cold acetone containing 0.07% (w/v) DTT for 1 h at −20°C. The protein precipitate was centrifuged again at 35,000 × *g* for 1 h (4°C) and washed three times with ice-cold acetone containing 0.07% (w/v) DTT. Protein was extracted by resuspending the final dry pellet in a dissolution buffer [8 M Urea, 50 mM triethyl ammonium bicarbonate (TEAB), pH 8]. The mixtures were heated in 30°C water bath for 1 h and subjected to ultrasonication at 50 W output with two bursts of 10 s each and the lysates were cooled on ice for 1 min between bursts. Following lysis, the protein extracts were clarified by centrifuging for 30 min at 25,000 × *g* (4°C). The supernatants were collected and quantified by Bradford assays, using BSA as a standard.

### Protein Digestion and Sample Cleanup

Protein samples (100 μg) were solved in 8 M urea (pH 8.5) in a volume ratio of 1:4 and added the solution to a 10 K ultrafiltration tube, centrifuged at 12,000 × *g* at 4°C for 15 min. The 100 mM iodoacetamide (IAA) was then added to the protein supernatant, and the mixture was incubated for 30 min in the dark. The proteins were then diluted 3-folds using 50 mM NH_4_HCO_3_ and digested with trypsin (100:1) for 20 h at 37°C. Trypsin digestion was stopped by the addition of NH_4_HCO_3_. After centrifugating at 12,000 × *g* for 20 min, the supernatants were collected and were then desalted with C18 spin tips and freeze-dried. All prepared samples were stored at −80°C until LC–MS/MS analysis.

### Liquid Chromatography–Mass Spectrometry Analyses

Liquid chromatography–mass spectrometry (LC–MS/MS) analysis was performed as previously described with some modifications (Cao et al., 2016). Each of the dried fractions was dissolved in 20 μL of 0.1% (v/v) formic acid and centrifuged at 20,000 × *g* for 10 min. The final concentration of the peptide solution was 0.4 μg/μL and the peptide (5 μL) was injected onto the trap column with a flow rate of 10 μL/min for 2 min using a Thermo Scientific Orbitrap Elite. The trap was equilibrated at a maximum pressure of 500 bar for 12 μL followed by column equilibration at a maximum of 500 bar for 3 μL before starting gradient elution of column. The peptide samples were subsequently eluted with a five-step linear gradient of A/B mixture (A: ddH_2_O with 0.1% formic acid, B: ACN with 0.1% formic acid): 0–10 min, 3–8% B; 10–120 min, 8–20% B; 120–137 min, 20–30% B; 137–143 min, 30–90% B; 143–150 min, 90% B. The column flow was maintained as 250 nL/min. The chromatographic system was composed of a trapping column (75 μm × 2 cm, nanoviper, C18, 3 μM, 100 A) and an analytical column (50 μm × 15 cm, nanoviper, C18, 2 μM, 100 A). Data collection was performed using Theromo Xcalibur Qual Browser and Proteome Discoverer 2.0 software.

## Results

### Identification and Phylogenetic Analysis of *RALF* Genes in *Brassica napus* Genome

**FIGURE 1:**
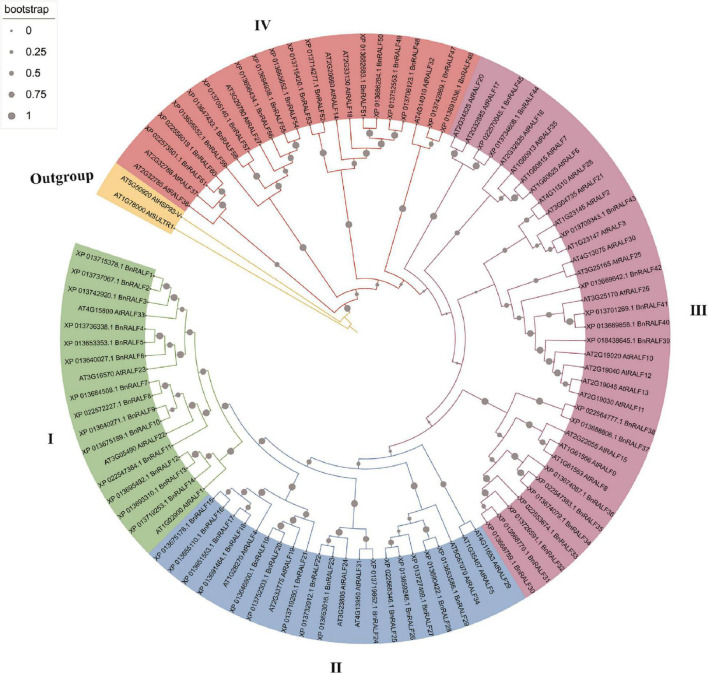
Phylogenetic tree of *B. napus* and *A. thaliana* RALF proteins. Full-length protein sequences were aligned using MUSCLE. The phylogenetic tree was constructed employing the UPGMA method with 1,000 bootstrap values in MEGA-X software, and was optimized with the iTOL online tool. The clades were marked with different colors. The gray filled circles in the branch indicate bootstrap value.

To investigate *RALF* genes in oilseed rape, a BLASTp search was performed against *B. napus* genome in NCBI database (see text footnote 2) using well-characterized *Arabidopsis* RALF protein sequences as query, which resulted in identification of 61 potential RALF sequences in the genome of *B. napus* (Table 1). To understand the evolutionary relationship of the RALFs, we constructed a phylogenetic tree based on the full-length BnRALF protein sequences employing the UPGMA method along with their 37 *A. thaliana* RALF homologs and two outgroup protein sequences (AT1G78000 and AT5G50920) from *Arabidopsis* (Figure 1). According to the phylogenetic analysis, these total 98 RALF protein sequences could be separated into four major clades (clades I–IV), which contained in turn 18, 21, 36, and 22 members. Each clade included at least one oilseed rape and one *Arabidopsis* RALFs, indicating that all clades evolved before the divergence of these two cruciferous lineages. However, uneven divergence existed between clades. BnRALFs accounted for over 70% in clades I [77% (14/18)], II [71% (15/21)], and IV [76% (16/22)], while only 44% (16/36) in clade III (Figure 1).

**TABLE 1:** List of BnRALFs identified in this study.

Gene name	Clade category	Accession no.	Predicted protein							
			Peptide length (aa)	Signal peptide length (aa)	pI	MW (Da)	Mature peptide length (aa)	RRXL domain	XIXY domain	Conserved cysteine residues
BnRALF1	I	XP_013715378.1	111	20	9.18	12187.93	53	RRIL	YISY	CCCC
BnRALF2		XP_013737067.1	111	–	8.99	12259.99	53	RRIL	YISY	CCCC
BnRALF3		XP_013742920.1	111	20	9.2	12221.95	53	RRIL	YISY	CCCC
BnRALF4		XP_013736338.1	133	29	9.07	14557.59	53	RRML	YISY	CCCC
BnRALF5		XP_013653353.1	133	28	9.09	14488.45	53	RRML	YISY	CCCC
BnRALF6		XP_013640027.1	127	27	9.3	13897.78	53	RRML	YISY	CCCC
BnRALF7		XP_013684508.1	118	22	8.52	12788.46	53	RRIL	YVSY	CCCC
BnRALF8		XP_022572227.1	118	22	9.03	12924.65	53	RRIL	YVSY	CCCC
BnRALF9		XP_013640271.1	119	22	9.35	12980.76	53	RRIL	YVSY	CCCC
BnRALF10		XP_013675189.1	119	22	9.03	13066.81	53	RRIL	YVSY	CCCC
BnRALF11		XP_022547384.1	116	22	9.35	12717.46	53	RRIL	YVSY	CCCC
BnRALF12		XP_013695492.1	121	25	7.57	13223.92	53	RRIL	YISY	CCCC
BnRALF13		XP_013693310.1	121	25	6.7	13188.83	53	RRIL	YISY	CCCC
BnRALF14		XP_013719253.1	150	–	9.46	16785.24	53	RRIL	YISY	CCCC
BnRALF15	II	XP_013675176.1	111	24	9.9	12733.78	57	RRQL	YIGY	CCCC
BnRALF16		XP_013655110.1	111	24	9.85	12705.68	57	RRQL	YIGY	CCCC
BnRALF17		XP_013651553.1	111	23	9.79	12658.62	57	RRQL	YIGY	CCCC
BnRALF18		XP_013691464.1	111	23	9.79	12644.59	57	RRQL	YIGY	CCCC
BnRALF19		XP_013646800.1	110	19	9.94	12441.44	57	RRQL	YISY	CCCC
BnRALF20		XP_013752303.1	110	19	9.93	12495.47	57	RRQL	YISY	CCCC
BnRALF21		XP_013719280.1	109	22	9.03	12444.34	56	RRQL	YISY	CCCC
BnRALF22		XP_013732912.1	151	–	9.16	16701.23	60	RRVM	YISY	CCCC
BnRALF23		XP_013653016.1	117	21	8.78	13156.16	60	RRVM	YISY	CCCC
BnRALF24		XP_013719862.1	110	22	5.61	12417.2	60	RRVL	YIGY	CCCC
BnRALF25		XP_022565346.1	122	23	9.5	13852.71	60	RRSL	YISY	CCCC
BnRALF26		XP_013659246.1	123	24	9.3	13961.9	60	RRSL	YISY	CCCC
BnRALF27		XP_013727469.1	130	24	6.41	14900.81	60	RRSL	YISY	CCCC
BnRALF28		XP_013690422.1	129	25	7.7	14405.27	60	RRSL	YISY	CCCC
BnRALF29		XP_013653586.1	129	26	7.7	14385.28	60	RRSL	YISY	CCCC
BnRALF30	III	XP_013668759.1	79	22	7.71	8775.19	57	–	YINY	CCCC
BnRALF31		XP_013668770.1	79	22	7.69	8725.12	57	–	YINY	CCCC
BnRALF32		XP_013726591.1	79	22	8.41	8763.18	57	–	YINY	CCCC
BnRALF33		XP_022553674.1	75	22	6.81	8236.44	53	–	YINY	CC–
BnRALF34		XP_013674079.1	75	27	9.34	8502.79	48	–	YINY	CCCC
BnRALF35		XP_022547383.1	75	27	9.34	8502.79	48	–	YINY	CCCC
BnRALF36		XP_013674087.1	75	26	8.8	8356.6	49	–	YIDY	CCCC
BnRALF37		XP_013688806.1	84	27	8.75	9305.84	57	–	YIDY	CCCC
BnRALF38		XP_022564777.1	137	27	8.75	15304.82	110	–	YIDY	CCCC
BnRALF39		XP_018438645.1	72	22	9.3	7823.27	50	–	YIDF	CCCC
BnRALF40		XP_013669858.1	74	22	8.85	7955.43	52	–	YLSP	CCCC
BnRALF41		XP_013701269.1	74	22	9.06	7972.38	52	–	YLSP	CCCC
BnRALF42		XP_013669642.1	79	24	7.67	8729.16	55	–	YLDP	CCCC
BnRALF43		XP_013709343.1	102	26	9.08	11271.83	76	–	YIGY	CCCC
BnRALF44		XP_013734608.1	67	25	8.35	6758.87	42	–	NIGN	–CC
BnRALF45		XP_022570045.1	67	24	8.27	6784.92	43	–	NIGG	CCCC
BnRALF46	IV	XP_013691036.1	117	22	7.59	13105.74	95	–	TLSY	CCCC
BnRALF47		XP_013742869.1	119	22	8.25	13460.11	97	–	TLSY	CCCC
BnRALF48		XP_013706123.1	105	24	9.27	11870.76	49	RRVL	YINY	–CC
BnRALF49		XP_013752553.1	106	25	9.54	11964.94	49	RRVL	YINY	–CC
BnRALF50		XP_013686284.1	102	24	7.63	11638.48	49	RRIL	YINY	–CC
BnRALF51		XP_013682683.1	103	24	8.31	11814.69	79	–	YINY	–CC
BnRALF52		XP_013714277.1	104	24	9.41	11811.87	80	–	YIKY	–CC
BnRALF53		XP_013715420.1	104	24	9.44	11751.82	80	–	YINY	–CC
BnRALF54		XP_013665652.1	115	23	9.54	12282.08	51	RRIL	KLSY	CCCC
BnRALF55		XP_013694938.1	117	25	9.5	12645.49	51	RRIL	KLSY	CCCC
BnRALF56		XP_013696434.1	115	24	9.05	12338.05	51	RRVL	KLSY	CCCC
BnRALF57		XP_013705140.1	115	23	9.3	12333.29	53	RRIL	KLSY	CCCC
BnRALF58		XP_013647433.1	115	25	9.32	12217.12	53	RRIL	NLGY	CCCC
BnRALF59		XP_013695552.1	112	24	8.59	12190.07	50	RRIL	VISP	CCCC
BnRALF60		XP_022556019.1	62	31	9.3	6925.36	31	–	VISY	CC–
BnRALF61		XP_022573501.1	63	31	9.3	7032.56	32	–	IISY	CC–

*“–”: None.*

The retrieved BnRALF protein sequences were further analyzed for physico-chemical properties (Table 1). These 61 BnRALF proteins displayed diversities in full length, signal peptide length, theoretical isoelectric point (pI), molecular weight (MW) and putative mature peptide length. Specifically, the full length of the 61 BnRALF protein sequences varied from 62 to 151 amino acids (aa), among which the majority of members in clade III were short with a full-length of less than 100 aa. Additionally, 93% (57/61) RALF proteins exhibited a pI higher than 7 and 90% (55/61) contained a signal peptide, the hallmark of post-translationally modified secreted peptide precursors, indicating that these proteins mainly function outside the cell.

### Conserved Motifs of BnRALF Proteins

**FIGURE 2:**
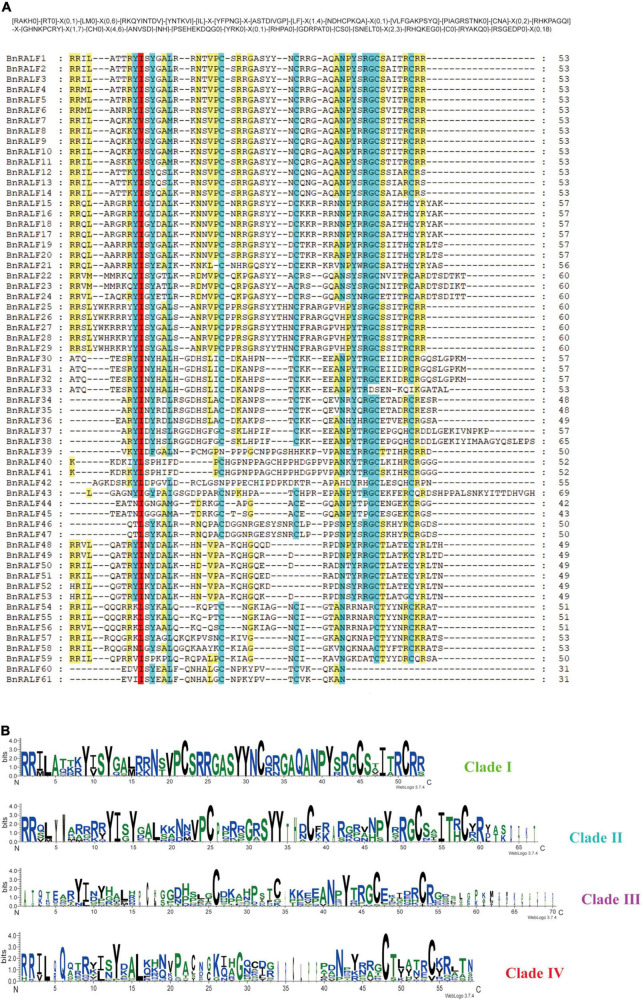
Conserved motifs of *B. napus* RALF proteins. **(A)** Multiple sequence alignment of BnRALF proteins for conserved motifs. Alignment was carried out using ClustalW and presented by GeneDoc. Residues with various conservation percentage were highlighted in different colors: 100% in red; 80∼100% in blue, 60∼80% in yellow. The square bracket ‘[ ]’ indicates the amino acids allowed in this position of motif; A ‘X’ represents any amino acid, while the round bracket ‘()’ denotes the number of amino acids. **(B)** Residue conservation of BnRALFs within the clades. The group-wise sequence logos showing the amino acid bias of the RALF mature peptides were generated from the WebLogo3 analysis. *Y*-axis expresses the bit score of each position in the sequence and the group names are indicated on the right.

To further characterize BnRALFs, we performed mature peptide sequence alignment using GeneDoc and motif prediction employing MEME tool (Bailey et al., 2009). The total sequence alignment and group-wide conservative sequences were shown in Figures 2A,B, respectively. The results showed that clades I, II, and IV all possessed the conserved RRXL (X represents any amino acid) motif, which is a protease cleavage site for the mature peptide process (Srivastava et al., 2009). Remarkably, this motif was entirely absent across the 16 clade III BnRALF proteins (Table 1 and Figure 2A). Given that the length of these proteins were already similar to that of mature proteins of other clades, these data implied that BnRALFs of the clade III likely required no further cleaving processing. This was likely also the case for two clade IV BnRALFs, BnRALF61 and BnRALF62. Uniquely, five BnRALFs of clade IV, BnRALF46, BnRALF47, BnRALF51, BnRALF52 and BnRALF53, did not harbor canonical RRXL motif either. They were comprised of 79–97 aa, which were much longer than the general size of mature BnRALF peptides (less than 60 aa). This indicated that these five BnRALFs required further maturation processing and were likely cleaved in a RRXL-independent mechanism.

The YISY motif, which is essential for activity and receptor binding of RALFs (Pearce et al., 2010; Xiao et al., 2019), existed in different forms in a clade-dependent manner, canonical YISY and its variant YVSY in clade I; canonical YISY and its variant YIGY in clade II; variants YIN/DY and YLS/DP dominated in clade III, while variants YINY and KLSY dominated in clade IV (Table 1 and Figure 2A). Whether the variants are as functional as the canonical YISY awaits further experimental verification.

Fifty-one out of 61 BnRALFs carried four conserved cysteines, which are supposed to be folded into intramolecular disulfide bridges for functions of RALFs (Pearce et al., 2001; Moussu et al., 2020; Zhang X. et al., 2020). The remaining 10 BnRALFs were distributed in the clades III and IV. They contained two cysteine residues at the first and second positions, or the third and fourth (Table 1 and Figure 2A), which may form one intramolecular disulfide bond.

Additionally, BnRALF38 and BnRALF43 of clade III contained a 53aa and 25aa extension at their C termini (Table 1 and Figure 2A). These extended sequences did not possess known motifs based on prediction analyses using SMART and Pfam tools. Whether these sequences play a role in the functions of these BnRALFs requires further study.

Collectively, BnRALF sequences are generally conserved within clades but diverse among clades, suggesting that they might be widely involved in a variety of biological processes.

### Expression Patterns of *BnRALF* Genes in Response to *Sclerotinia sclerotiorum* Infection and BnPep5 and SsNLP1 Treatments

**FIGURE 3:**
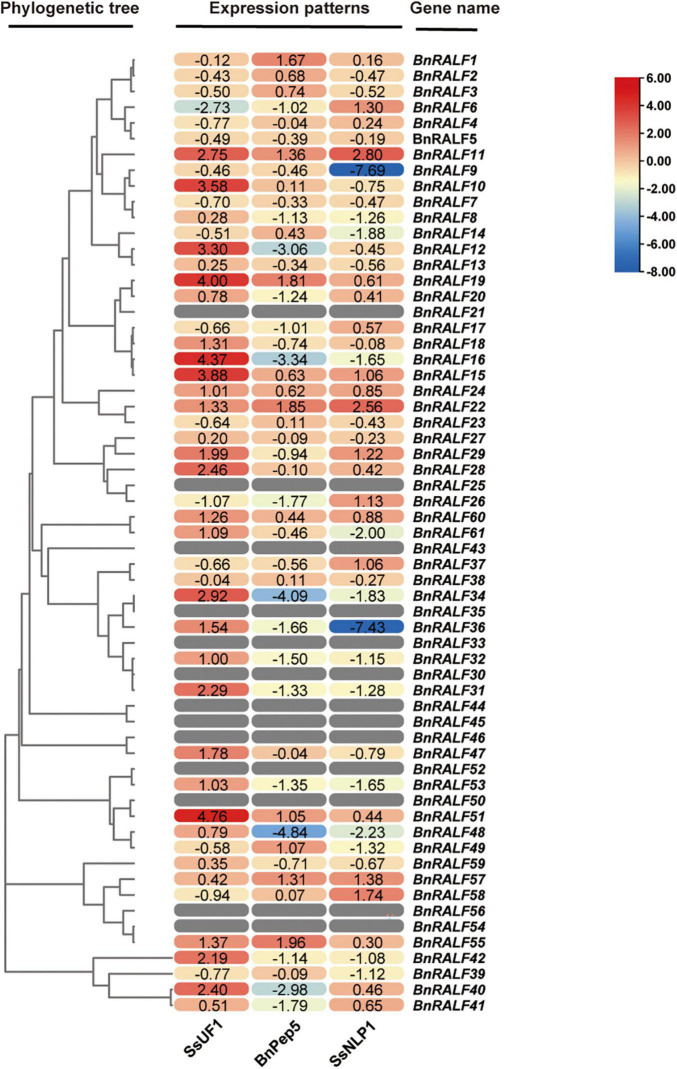
Expression patterns of the whole *BnRALF* gene family in response to different stimuli. The heatmap was constructed by TBtools to present expression patterns of the 61 *BnRALF* genes in oilseed rape leaves inoculated with *S. sclerotiorum* strain UF1 (SsUF1) or infiltrated with 0.2 μM BnPep5 (*B. napus* plant elicitor peptide 5) or 1 μM SsNLP1 [*S. sclerotiorum* ethylene-inducing peptide 1 (SsNep1)-like peptide 1] peptide solution (*n* = 3 for each group). PDA plug mock inoculation was served as the control for the SsUF1 inoculation, while ddH_2_O infiltration was conducted as the control for the BnPep5 and SsNLP1 infiltration treatments. The expression levels from low to high are indicated by a change in color from blue to red. The numbers in the figure are represented as log(2)-fold change of treated samples versus mock control. These experiments were performed three times, each yielding similar results.

To obtain hints for functions of *BnRALF* genes in immunity, we systematically examined the expression patterns of all 61 *BnRALF* genes in response to pathogen (*S. sclerotiorum*) infection and DAMP (BnPep5) and PAMP (SsNLP1) treatments via quantitative real-time PCR (qRT-PCR) analysis. BnPep5, a *B. napus* plant elicitor peptide, was identified by our lab via BLASTp search using well-characterized Arabidopsis Peps as query sequences (unpublished data), and SsNLP1 was identified by our lab from *S. sclerotiorum* necrosis and ethylene-inducing peptide 1 (SsNep1) (Bashi et al., 2010, our unpublished data). Both BnPep5 and SsNLP1 could elicit immunity to *S. sclerotiorum* in *B. napus* (unpublished data). To compare the *BnRALF* expression patterns in response to different treatments, we converted the qRT-PCR gene expression values into a heatmap on the basis of the log_2_-fold change-derived data (Figure 3). Comparison of *BnRALF* expression profiles in response to *S. sclerotiorum* at 0 and 7 h post inoculation (hpi) showed that the expression of most *BnRALF*s were up-regulated by *S. sclerotiorum* infection. Among them, the expression level of *BnRALF10*, *−15*, *−16*, *−19*, and *−51* was most up-regulated by over 10-fold. *BnRALF6* was the only one whose expression was significantly down-regulated, by 6.6 folds (Figure 3). This result suggested that these *BnRALF* genes may be involved in the regulation of the interaction between *B. napus* and *S. sclerotiorum*.

In contrast, more *BnRALF* genes were expressionally down-regulated in response to DAMP and PAMP treatments. At 2 h post treatment with 200 nM BnPep5, only *BnRALF1*, *−19*, *−22*, and *−55* were expressionally up-regulated by over threefold, while *BnRALF12*, *−16*, *−34*, *−40*, and *−48* were expressionally decreased by 5∼28 folds. At 2 h post treatment with 1 μM SsNLP1, expression of *BnRALF9* and *BnRALF36* were extremely reduced by over 100-fold, while that of *BnRALF11* and *BnRALF22* were enhanced by fivefold and sevenfold, respectively (Figure 3). These genes were strongly responsive to DAMP/PAMP treatments and thus may play an important role in plant immunity to *S. sclerotiorum*.

The expression of 13 *BnRALF* genes, *BnRALF21*, *−25*, *−30*, *−33*, *−35*, *−43*, *−44*, *−45*, *−46*, *−50*, *−52*, *−54*, and *−56* could not be detected no matter under normal growth conditions or after treated with three stimuli in our assays (Figure 3), implying their expression was extremely low, if any in leaves.

Taken together, the expression patterns of the whole BnRALF family indicate that BnRALF members might be differentially involved in immunity to *S. sclerotiorum*.

### BnRALF10 Elicits Resistance to *Sclerotinia sclerotiorum*

**FIGURE 4:**
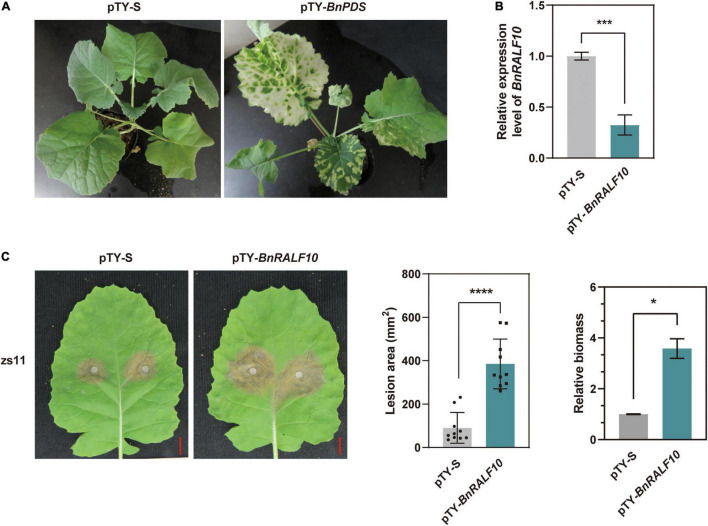
Silencing of *BnRALF10* reduced resistance to *S. sclerotiorum* in oilseed rape. **(A)** Photobleaching phenotype of *BnPDS* silencing in oilseed rape employing pTY-based VIGS. Empty vector pTY-S was used as a control. **(B)** Efficient silencing of *BnRALF10* in leaves as manifested by the dramatically reduced level of *BnRALF10* transcript, which was detected by qRT-PCR analysis using *B. napus* actin (*BnActin7*) as reference gene. **(C)** Representative disease symptoms (left), lesion areas (middle) and fungal biomass (right) of *BnRALF10*-silenced leaves at 24 h post inoculation with *S. sclerotiorum*. Scale bar: 1 cm. Data are statistically analyzed by Student’s *t*-test (*n* = 10) and shown as the mean ± SE. The asterisks indicate significant differences (*P ≤ 0.05, ***P ≤ 0.001, ****P ≤ 0.0001).

Our observation that the expression of *BnRALF10* was strongly up-regulated by 10-fold in response to *S. sclerotiorum* inoculation (Figure 3) and the reported fact that its *Arabidopsis* ortholog AtRALF22 stimulated immune responses such as ROS accumulation (Abarca et al., 2021) prompted us to perform functional analyses of *BnRALF10* in immunity to *S. sclerotiorum*. The *Turnip yellow mosaic virus* (TYMV)-based virus induced gene silencing (VIGS) technology was employed to knock-down *BnRALF10* in *B. napus*. A typical photobleaching symptom (caused by silence of the *BnPDS* gene) in *B. napus* plants bombarded with pTY-*BnPDS* was observed on the newly emerged leaves 14 days after treatment (Figure 4A), indicating that TYMV-mediated gene silencing was effective in *B. napus*. Meanwhile, the plants treated with pTY-S (empty vector) showed normal growth (Figure 4A), suggesting that the TYMV-based vector infection did not obviously affect plant vegetative growth. In plants bombarded with pTY-*BnRALF10*, the recombinant pTY vector containing both sense and antisense versions of a specific 40 bp fragment from the coding region of *BnRALF10* so that to form its self-hybridized palindromic fragment, *BnRALF10* expression was significantly reduced (Figure 4B). Additionally, expression level of the closest homologs of *BnRALF10* (*BnRALF8*, *BnRALF9*, *BnRALF11*), which were clustered in the same clade with *BnRALF10*, but not less close homologs of *BnRALF10* (*BnRALF12* and *BnRALF13*), which were not clustered in the same clade with *BnRALF10*, was also decreased in the pTY-*BnRALF10*-silence treated plants (Supplementary Figure 1). Compared with the plants treated with pTY-S, the oilseed rape plants with decreased *BnRALF10*-clade gene expression exhibited more severe necrosis, larger disease areas and increased *S. sclerotiorum* biomass (Figure 4C). This indicated that silencing of *BnRALF10* and its clade homologs increased susceptibility to *S. sclerotiorum* challenge.

**FIGURE 5:**
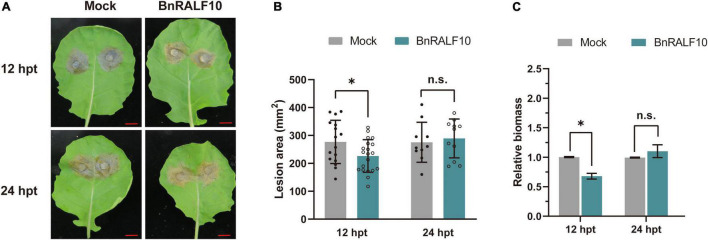
BnRALF10 peptide induced plant resistance to *S. sclerotiorum*. Representative disease symptoms **(A)**, lesion area **(B)** and fungal biomass statistical analysis **(C)** of oilseed rape leaves pretreated with 1 μM BnRALF10 peptide solution or ddH_2_O (Mock) at 12 h and 24 h post inoculation with *S. sclerotiorum*. Scale: 1 cm. Data are shown as the mean ± SE. The asterisks indicate significant differences from the control, as determined by one-way ANOVA (*n* = 7 leaves, n.s., not significant, *P ≤ 0.05). The experiments were repeated three times with similar results.

To further confirm the role of BnRALF10 in resistance to *S. sclerotiorum*. We synthesized the mature BnRALF10 peptide comprising the conserved YISY-motif and four conserved cysteines. The oilseed rape leaves were preinfiltrated with 1 μM BnRALF10 peptide solution, and were then challenged with *S. sclerotiorum* 12 h and 24 h later, respectively. BnRALF10 treatment for 12 h enhanced the resistance against *S. sclerotiorum*, as indicated by the weaker disease symptom and reduced lesion size in leaves treated with BnRALF10 compared to the control plant leaves (Figure 5). However, at 24 h post infiltration, the plants treated with BnRALF10 exhibited similar disease symptom and lesion size to the control plants (Figure 5). This result suggests that resistance elicited by BnRALF10 is not long-lasting.

Taken together, these results demonstrate that BnRALF10 stimulates resistance to *S. sclerotiorum* in *B. napus*.

### BnRALF10 Activates Plant Immune Responses

**FIGURE 6:**
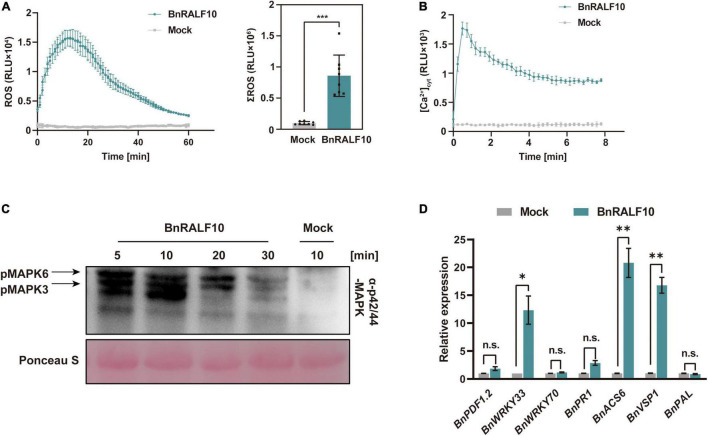
BnRALF10 elicited various immune responses. **(A)** Reactive oxygen species (ROS) measured in oilseed rape leaf disk assay after addition with 1 μM BnRALF10 peptide or water (Mock). Shown are the dynamics of ROS production (left) and their total amount (right) as mean values of total photon counts over 60 min. Data are shown as the mean ± SE (*n* = 6–8, ^***^P ≤ 0.001, Student’s *t*-test). RLU, relative light units. All experiments were repeated three times with similar results. **(B)** Dynamics in [Ca^2+^]_cyt_ stimulated by 1 μM BnRALF10 peptide in Arabidopsis. The 5-day-old seedlings expressing *Aequorin* gene were treated with solutions containing 1 μM BnRALF10 peptide or mock. Signals were recorded for 8 min after treatment. Data are shown as the mean ± SE (*n* = 8). **(C)** MAPK activation induced by BnRALF10. Seven-day-old seedlings were exposed to 1 μM BnRALF10 peptide for 5, 10, 20 or 30 min. Western blot analysis was performed with the phospho-p44/42 MAPK antibody. Ponceau S was used as loading control. **(D)** Transcription of defense responsive genes in *B. napus* leaves treated with 1 μM BnRALF10 for 1 h. Data are shown as the mean ± SE (*n* = 3, *P ≤ 0.05, ^**^P ≤ 0.01, Student’s *t*-test). n.s., not significant.

Given that *BnRALF10* expression was rapidly and strongly induced by *S. sclerotiorum*, and that BnRALF10 stimulated resistance against this pathogen, we inferred that BnPRORALF10 might encode a peptide that act as a DAMP. To confirm this, we analyzed the functions of BnRALF10 in activating early immune responses such as ROS production, [Ca^2+^]_cyt_ promotion, MAPK activation and defense gene expression promotion. L-012-based leaf disk assay demonstrated that supply with 1 μM BnRALF10 peptide strongly induced extracellular ROS accumulation (Figure 6A). Further, we used the transgenic *Arabidopsis* seedlings carrying the calcium reporter aequorin to measure [Ca^2+^]_cyt_ after treatment with 1 μM BnRALF10. A rapid [Ca^2+^]_cyt_ elevation was observed after BnRALF10 treatment (Figure 6B). Moreover, MAPK assay in *Arabidopsis* seedlings revealed that MAPK3 and MAPK6 was promptly activated upon stimulation with BnRALF10 (Figure 6C). In addition, we examined transcriptional induction of genes closely linked to defense. Expression of *BnWRKY33*, *BnACS6* and *BnVSP1* was strongly induced by 12∼21-fold at 1 h post BnRALF10 treatment, while that of *BnPDF1.2*, *BnWRKY70*, *BnPR1* and *BnPAL* was not obviously altered (Figure 6D).

Taken together, these results indicate that BnRALF10 elicits diverse immune responses as a DAMP in *B. napus*, and triggers DTI (DAMP-triggered immunity) against the necrotrophic pathogen *S. sclerotiorum*.

### BnFER Is a Receptor for BnRALF10

Holding ascertained that BnRALF10 modulates *S. sclerotiorum* resistance, we sought to investigate the molecular mechanism behind it. BnRALF10 is the close homolog of Arabidopsis RALF22 and RALF23, which are recognized by AtFER (Stegmann et al., 2017; Zhao et al., 2018). Therefore, we wondered whether the *B. napus* homolog of AtFER was the receptor of BnRALF10. To verify this, we identified BnFER and performed functional analyses for it. BLASTp search using AtFER protein sequence as query retrieved a *B. napus* homolog of AtFER (BnFER) in cultivar Zhongshuang 11 (ZS11) (Supplementary Figure 2). Domain composition analysis showed that like AtFER, BnFER consisted of two extracellular malectin domains, a transmembrane domain and an intracellular kinase domain (Supplementary Figure 2).

**FIGURE 7:**
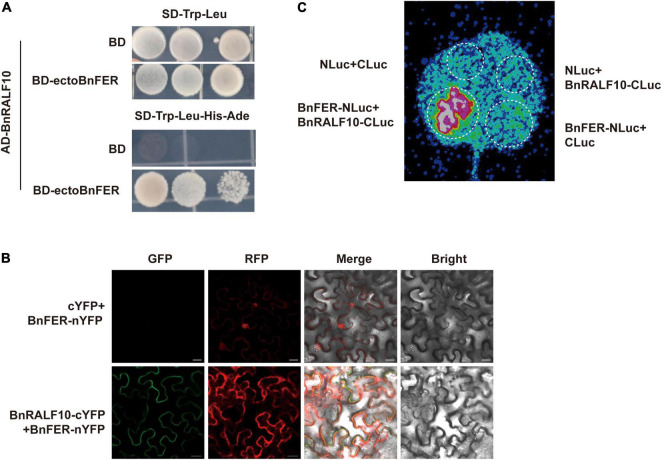
BnRALF10 physically interacted with BnFER. **(A)** BnRALF10 interacts with the extracellular domain of BnFER in yeast. Yeast (strain AH109) co-transformed with pGBKT7 (BD) or the BD-ectoBnFER bait vector and pGADT7 (AD)-BnRALF10 prey vector was grown on SD–Trp–Leu, SD–Trp–Leu–His–Ade plates for interaction confirmation. Only yeast co-transformed with BD-ectoBnFER and AD-BnRALF10 grew on the SD–Trp–Leu–His–Ade medium. BD + AD-BnRALF10 was used as negative control. **(B)** BiFC assay to detect BnRALF10–BnFER interaction in *N. benthamiana*. YFP fluorescence was detected in *N. benthamiana* leaves co-expressing BnRALF10-cYFP and BnFER-nYFP (Lower) but not in the control (cYFP + BnFER-nYFP; Upper). Scale bar: 20 μm. **(C)** LCI assay to monitor BnRALF10–BnFER interaction in *N. benthamiana*. LUC activity was detected in *N. benthamiana* leaves co-expressing BnRALF10-CLuc and BnFER-NLuc but not in the controls (BnRALF10-CLuc + NLuc, CLuc + BnFER-NLuc and CLuc + NLuc).

Next, we employed three methods to discern the physical interaction between BnRALF10 and BnFER. Y2H assay showed that BnRALF10 physically interacted with the extracellular domain of BnFER (ectoBnFER) in yeast (Figure 7A). We further conducted BiFC and LCI assay to validate the interaction between BnRALF10 and BnFER *in planta*. A strong Yellow Fluorescent Protein (YFP) signal was observed at the plasma membrane when BnFER-nYFP (BnFER fused with the N-terminal fragment of YFP) was co-expressed with BnRALF10-cYFP (BnRALF10 fused with the C-terminal fragment of YFP), whereas no YFP signal was detected in leaves expressing BnFER-nYFP and cYFP (Figure 7B). Moreover, co-expression of BnFER-NLuc (BnFER fused with the N-terminal fragment of Luciferase) with BnRALF10-CLuc (BnRALF10 fused with the C-terminal fragment of Luciferase), but not that of BnFER-NLuc and CLuc, NLuc and BnRALF10-CLuc, or NLuc and CLuc, resulted in strong LUC (Luciferase) activity (Figure 7C). These results verify that BnRALF10 indeed can interact with BnFER in the plasma membrane.

**FIGURE 8:**
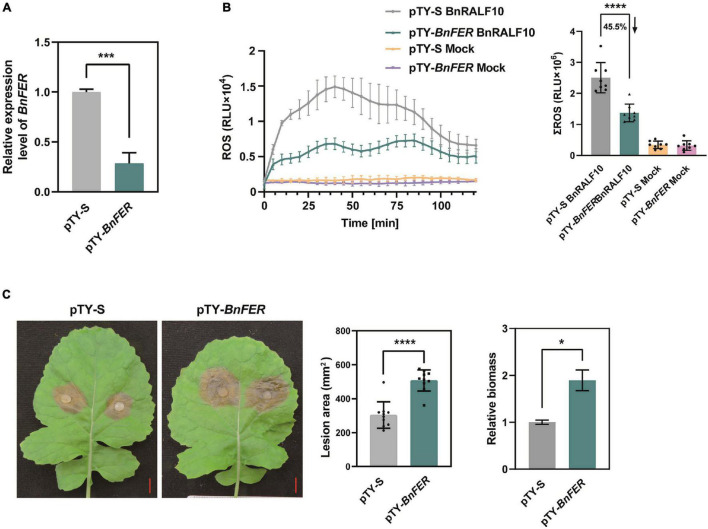
BnFER was required for BnRALF10 to function. **(A)** Efficient silencing of *BnFER* by pTY-*BnFER* treatment in rape leaves as manifested by the vastly reduced level of *BnFER* transcript, which was detected by qRT-PCR analysis using *B. napus* actin (*BnActin7*) as reference gene. Data are shown as the mean ± SE (*n* = 7, ***P ≤ 0.001, Student’s *t*-test). **(B)** Effect of *BnFER*-silencing on BnRALF10-induced ROS burst. ROS measured in rape leaf disks of *BnFER*-silenced and control plants after addition with 1 μM BnRALF10 peptide or water (mock). Shown are the dynamics of ROS production (left) and the integration (right) as mean values of total photon counts over 120 min. Data are shown as the mean ± SE (*n* = 8, ^****^P ≤ 0.0001, Student’s *t*-test). **(C)** Effect of *BnFER*-silencing on BnRALF10-induced resistance to *S. sclerotiorum*. Leaves of *BnFER*-silenced and control plants were inoculated with *S. sclerotiorum*. Disease symptoms (left), lesion area (middle) and fungal biomass in inoculated leaves (right) at 24 hpi were shown. Data are shown as the mean ± SE (*n* = 7, *P ≤ 0.05, ^****^P ≤ 0.0001, Student’s *t*-test).

Further, we investigated whether BnFER is involved in BnRALF10 signaling and defense against *S. sclerotiorum*. pTY-S-based VIGS technology was used to silence *BnFER* in *B. napus*. In VIGS plants treated with pTY-*BnFER*, *BnFER* expression was significantly reduced (Figure 8A). These rape plants with decreased expression of *BnFER* accumulated only 45.5% of BnRALF10-elicited ROS compared with pTY-S-treated control plants (Figure 8B), suggesting that BnFER is required for BnRALF10 to stimulate immune responses including oxidative burst. When inoculated with *S. sclerotiorum*, these *BnFER* knock-down plants exhibited more severe symptoms with significantly larger lesions and two-fold increased pathogen biomass compared to the control plants (Figure 8C), indicating that BnFER is required for BnRALF10 to elicit resistance to *S. sclerotiorum*.

Collectively, our results support BnFER to be a receptor for BnRALF10.

### Proteomic Analysis Reveals the Molecular Basis of BnRALF10-Induced Plant Immunity

**FIGURE 9:**
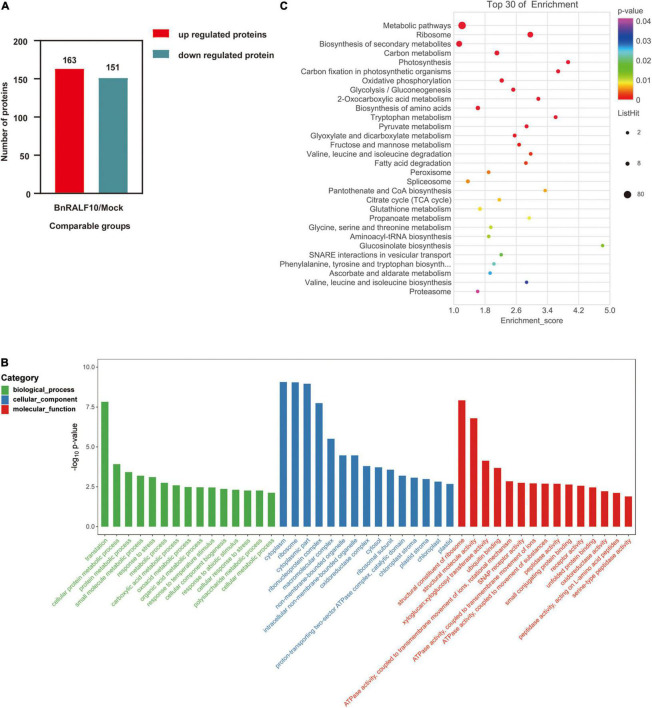
Functional categorization of proteins differentially expressed between oilseed rape leaves infiltrated with 1 μM BnRALF10 and ddH_2_O. The expression trend **(A)**, GO and KEGG analyses of differentially expressed proteins **(B,C)** (*p* < 0.05 and fold change >2 or <2) were presented. Leaves of 4-week-old oilseed rape plants were sampled at 4 h after infiltration with ddH_2_O and 1 μM BnRALF10, respectively. The bubble size represents the number of proteins, and the bubble color means the *p*-value of the significance. The pathway enrichment statistical analysis was performed by Fisher’s exact test. Thirty pathways showing the lowest *p*-value were selected for this analysis.

To reveal the possible mechanisms associated with the plant immunity stimulated by BnRALF10, we performed quantitative proteomic analysis to identify the proteins involved in this immunity. Total protein was extracted using the trichloroacetic acid (TCA)-acetone precipitation method for comparative proteomic analysis of the *B. napus* leaves at 4 h post infiltration with either 1 μM BnRALF10 or ddH_2_O as control. Principal components analysis (PCA) was performed to evaluate the test samples, and the result showed that the different groups of samples were well-distinguished, while three replicate samples of each group were clustered (Supplementary Figure 3), suggesting that the samples were highly qualified for proteomic analysis. We set the criteria for significantly differentially expressed proteins (DEPs) as that *p* < 0.05 and fold change >2 or <2 in three biological replicates. Based on these criteria, we identified 314 DEPs differentially expressed between leaves infiltrated with 1 μM BnRALF10 and ddH_2_O (Supplementary Table 2), including 163 up-regulated and 151 down-regulated proteins (Figure 9A).

GO annotation and KEGG pathways for DEPs were analyzed to understand the BnRALF10-stimulated response events and downstream biological processes. GO analysis showed that these DEPs belonged to 460 GO terms for 170 biological processes, 179 cellular components and 111 molecular functions (Supplementary Table 3). Among the main categories with the lowest *p* value in biological process (BP), the interesting terms included response to stress, response to abiotic stimulus and cellular response to stress, while the interesting molecular function (MF) comprised ubiquitin binding, ATPase activity coupled to transmembrane movement of ions and substances, SNAP receptor activity, receptor activity and peptidase activity (Figure 9B). KEGG pathway annotation analysis revealed that the DEPs were associated with 87 pathways (Supplementary Table 4). Among the 30 pathways with the lowest *p* value, the interesting pathways were the metabolic pathway, biosynthesis of secondary metabolites, peroxisome, glutathione metabolism, glucosinolate biosynthesis and proteasome (Figure 9C).

Intriguingly, dozens of DEPs have been reported to play a role in defense against various pathogens including *S. sclerotiorum* (Table 2). Here, we tentatively term the BnRALF10-elicited defense proteins as RED proteins. Among the REDs were a group of proteins involved in ROS generation and homeostasis, including ROS generator peroxisomal (*S*)-2-hydroxy-acid oxidase GLO2, and oxidoreduction regulators such as two glutathione *S*-transferase (GST) proteins and cytochrome P450 71A1. Remarkably, all these REDs were reported to function in plant defense against *S. sclerotiorum* (Table 2), demonstrating that BnRALF10 might tune GLOs- and GSTs-mediated ROS accumulation for immunity to *S. sclerotiorum*.

**TABLE 2:** List of functionally proved BnRALF10-elicited defense (RED) proteins.

Locus/protein ID	Log_2_FC (BnRALF10/ddH_2_O)	Annotation	Reported defense against (pathogens)	References
BnaA04g26460D/A0A078HJQ0	+	Remorin	*Sclerotinia sclerotiorum Setosphaeria turcica*	Jamann et al., 2016; Zhang Y. et al., 2021
			*Potato virus X*	Perraki et al., 2018
			*Puccinia polysora*	Wang S. et al., 2019
			*Xanthomonas campestris*	Ma et al., 2022
			*Rice stripe virus*	Fu et al., 2018
BnaAnng19920D/A0A078JH01	–	Profilin-5 (PRF5)	*Pseudomonas syringae*	Sun et al., 2018
BnaC09g33170D/A0A078H2M7	−1.35	Profilin-5 isoform X2	*Pseudomonas syringae*	Sun et al., 2018
BnaC09g39400D/A0A078CXF9	+	Peroxisomal (*S*)-2-hydroxy-acid oxidase GLO2	*Sclerotinia sclerotiorum* *Pseudomonas syringae*	Rojas et al., 2012; Xu Y. P. et al., 2018
BnaC03g30870D/A0A078FU46	+	Glutathione *S*-transferase F3-like	*Sclerotinia sclerotiorum*	Zou et al., 2021
BnaC03g41180D/A0A078GC51	+	Glutathione *S*-transferase DHAR1	*Sclerotinia sclerotiorum*	Cao et al., 2020
BnaA03g58610D/A0A078JG98	+	Cytochrome P450 71A1-like	*Sclerotinia sclerotiorum*	Zhang Y. et al., 2021
BnaA08g06260D/A0A078HYS1	–	Annexin D1	*Golovinomyces cichoracearum*	Zhao et al., 2021
BnaA09g43480D/A0A078FL33	+	Synaptotagmin-1	Many viruses *Golovinomyces cichoracearum*	Levy et al., 2015; Kim et al., 2016
BnaC03g73490D/A0A078JTQ4	+	Syntaxin-121 (PEN1)-like	*Golovinomyces cichoracearum*	Johansson et al., 2014
BnaA02g07110D/A0A078EQE4	1.65	Non-specific lipid-transfer protein 4-like	*Botrytis cinerea Sclerospora graminicola*	Bi et al., 1999; Manjula et al., 2015
BnaA04g21650D/A0A078G0A0	1.71	Non-specific lipid-transfer protein 1	*Botrytis cinerea Sclerospora graminicola*	Bi et al., 1999; Manjula et al., 2015
BnaA03g18750D/A0A078F2P1	−1.45	Mannose-1-phosphate guanylyltransferase 1	*Pseudomonas syringae*	Pavet et al., 2005
BnaA03g10790D/A0A078HG60 BnaA05g29880D/A0A078HDS6	+ –	Heat shock protein 90-2-like Heat shock protein 90-6	*Pseudomonas syringae*	Lopez et al., 2019
			*Tobacco mosaic virus*	Qian et al., 2018
BnaC04g25190D/A0A078HZM2	–	Heat shock 70 kDa protein 15	*Turnip mosaic virus*	Jungkunz et al., 2011
BnaA04g29360D/A0A078ITZ9	+	Amino acid transporter AVT6D	*Heterodera glycines*	Guo et al., 2019
BnaA05g30940D/A0A078GDT7	–	ISWI chromatin-remodeling complex ATPase CHR11-like	*Pseudomonas syringae*	Pardal et al., 2021
BnaC04g52530D/A0A078ILD9	+	Multiprotein-bridging factor 1a-like	*Botrytis cinerea*	Kim et al., 2007
BnaC02g33940D/A0A078HI06	+	Nicotinate-nucleotide pyrophosphorylase	*Pseudomonas syringae*	Petriacq et al., 2012
BnaC02g22640D/A0A078I7J9	+	Cytosolic sulfotransferase 16-like (ST5a)	*Sclerotinia sclerotiorum*	Piotrowski et al., 2004
BnaCnng41810D/A0A078JEQ2	–	Subtilisin-like protease SBT1.7	*Plasmopara viticola*	Figueiredo et al., 2016
BnaCnng55020D/A0A078JPY4	–	Subtilisin-like protease	*Plasmopara viticola*	Figueiredo et al., 2016
			*Pseudomonas syringae*	Jordá et al., 1999
BnaC01g21920D/A0A078H9L4	−1.28	Probable cysteine protease RD19C	*Ralstonia solanacearum*	Bernoux et al., 2008
BnaC01g36130D/A0A078G5U7	–	Lectin-like protein LEC	*Sclerotinia sclerotiorum*	Wang et al., 2021
			*Pseudomonas syringae*, *Phytophthora parasitica*, *Turnip mosaic virus*	Ma et al., 2010
BnaC02g32860D/A0A078HYK6	+	Syntaxin-22-like	*Tobacco mosaic virus*	Ibrahim et al., 2020
			*Meloidogyne incognita*	Zhu et al., 2019

*“+”: Proteins detected in BnRALF10- but not ddH_2_O-infiltrated leaves. “–”: Proteins detected in ddH_2_O- but not BnRALF10-infiltrated leaves.*

A remorin (REM) and two profilins (PRFs) are also among the RED proteins. REM positively while PRFs negatively regulate formin-mediated actin assembly to create membrane compartments for immune receptor complex formation and promote immune signal transduction thereby elicit plant immunity (Sun et al., 2018; Ma et al., 2022). Coincidentally, BnRALF10 increased REM accumulation while decreased PRFs accumulation (Table 2), and they are involved in plant immunity against diverse pathogens including *S. sclerotiorum* (Table 2), revealing that BnRALF10 likely fine-tunes REM- and PRFs-mediated actin cytoskeleton to stimulate plant immunity.

Some REDs exhibit Ca^2+^-dependent functions in plant defense. Among them were an annexin (ANN) and a synaptotagmin (SYT). AtSYT1 physically interacts with AtSYP121/PEN1, another RED, to modulate exocytosis for localization of immune proteins to plasma membrane (PM). AtANN8 and AtSYT1 regulate plant immunity to fungal and viral pathogens (Levy et al., 2015; Kim et al., 2016; Zhao et al., 2021). BnRALF10 strikingly changed the accumulation level of a variety of calcium sensors such as ANNs, SYTs, IQ-domain proteins (IQDs), CBL-interacting serine/threonine-protein kinase 4 (CIPK4), E3 ubiquitin-protein ligase HOS1-like protein, 14-3-3-like protein GF14 omega, serine/threonine-protein kinase STN7 and syntaxin 22-like protein (Supplementary Table 2). This result implies that BnRALF10 might rely heavily on calcium pathway to fine tune plant immunity.

Other RED proteins included two transcriptional regulators CHR11 (chromatin-remodeling protein 11) and MBF1a (multiprotein bridging factor 1a), two LTPs (lipid transfer proteins), three HSPs (heat shock proteins), two SBTs (subtilisin-like proteases), cysteine protease RD19C, sulfotransferase ST5a, mannose-1-phosphate guanylyltransferase 1, glucan endo-1,3-beta-glucosidase, syntaxin-22-like and lectin-like proteins (Table 2).

Collectively, the proteomic analysis results reveal that BnRALF10 likely modulates the abundance of RED proteins to fine-tune plant immunity against pathogens including *S. sclerotiorum*.

## Discussion

### Rapid Alkalinization Factor Family in Oilseed Rape

Genome-wide identification of RALFs in cruciferous species has been performed in *Arabidopsis thaliana* and *Brassica rapa*. Consequently, 37 and 32 *RALF* genes were identified in *A. thaliana* and *B. rapa*, respectively (Campbell and Turner, 2017; Abarca et al., 2021). In the present study, we identified 61 *RALF* genes in *B. napus*, another cruciferous species (Table 1). The RALF gene copy number in *B. napus* is much higher than that in *A. thaliana* and *B. rapa* although the three species belong to the same family. *B. napus* is an allotetraploid from crossing between *B. oleracea* and *B. rapa*, followed by chromosome doubling (Chalhoub et al., 2014). The copy number of *RALF* genes in *B. napus* almost doubles that in *B. rapa*, implying that the divergence of the RALF family may occurred as chromosome doubling. *Brassica* species underwent whole genome triplication (WGT) after the divergence of the *Brassica* ancestor and the genus *Arabidopsis*, which includes *A. thaliana* (Cheng et al., 2017), while the number of *RALF* genes in *B. napus* is not triple of that in *A. thaliana*. Considering that *B. napus* is tetraploid while *A. thaliana* is diploid, it is likely that the evolution of *RALF* genes was caused by segmental duplication.

Phylogenetic analysis for RALFs in *B. napus* and *A. thaliana* showed that these 98 RALFs diverged into four clades (Table 1 and Figure 1). Among them, clades I and II can be considered the canonical RALFs, since they contain the features previously depicted to be characteristic of the RALF family, including the N-terminal signal peptide cleavage site, S1P cleavage site RRXL, YISY motif and C-terminal four conserved cysteines (Campbell and Turner, 2017). *BnRALF* genes which strongly responded to *S. sclerotiorum* inoculation belonged to these two clades except *BnRALF51* (Figure 3), indicating that the canonical RALF proteins may play a major role in the resistance to *S. sclerotiorum*. As matter of fact, BnRALF10, a member of clade I, indeed induced the resistance to *S. sclerotiorum* (Figures 4, 5), further supporting the important roles of canonical BnRALFs in immunity against the necrotrophic pathogen *S. sclerotiorum*.

RRXL dibasic site has been reported to be recognized by S1P and necessary for maturing of PRORALFs (Matos et al., 2008; Stegmann et al., 2017). Twenty-three BnRALFs lacked canonical RRXL site (Table 1 and Figure 2). They may execute either no further processing or one independent of S1P. All 16 clade III and two clade IV BnRALF proteins were significantly smaller than typical BnRALFs of clades I and II, rather similar to their mature peptides, suggesting that these 18 BnRALFs likely undergo no further cleaving processing. The remaining five, BnRALFs 46, 47, 51, 52 and 53, all belonging to clade IV, have a size similar to typical BnRALFs, indicating that they might perform further maturation processing and were likely cleaved in a RRXL-independent mechanism. Noticeably, they carry a single R in the corresponding RRXL position of typical BnRALFs, HRIL in BnRALF52 and BnRALF53, RKIL in BnRALF51, QRFT in BnRALF46 and QRLT in BnRALF47. Interestingly, monobasic cleavage sites have been found in proteins from vertebrates and insects (Veenstra, 2000), suggesting that these BnRALFs might be cleaved by proteases recognizing monobasic cleavage sites. Whether this is indeed the case as in vertebrates and insects awaits experimental verification. The S1P cleavage site seems to be important for the physiological function of RALFs. *Arabidopsis* RALF23 and its relative RALF33, with RRXL sites, are proteolytically cleaved by S1P and play negative roles in plant immunity, while non-S1P cleaved RALF peptide AtRALF17 is devoid of a propeptide region, positively regulates plant immunity (Stegmann et al., 2017). AtRALF22 regulates salt stress in a S1P-dependent way (Zhao et al., 2018). BnRALF10 contains the conserved RRXL site (Table 1). BLASTp search using AtS1P as query retrieved three *B. napus* orthologs of AtS1P (BnS1Ps) (Supplementary Figure 4A), which have the same domain composition with AtS1P, both containing the peptidase_S8 domain (Supplementary Figure 4B). The high similarity between AtS1P with the putative BnS1Ps implies that they may share the same functions. Together, it is highly possible that the precursor of BnRALF10 is processed in the RRXL site to release a C-terminal mature peptide by the putative BnS1Ps. Whether the maturation of BnRALF10 and its role in inducing resistance to *S. sclerotiorum* indeed depends on the S1P cleavage site and the putative BnS1Ps, is worth further study.

Rapid alkalinization factor was first associated with the plant defense response for its ability in MAPK activation (Pearce et al., 2001). Later, it was shown to be able to elicit ROS burst (Thynne et al., 2017), cytoplasmic Ca^2+^ spikes and apoplastic pH ascending in *Arabidopsis* root cells (Gjetting et al., 2020), which are hallmarks of defense activation by plants. Here we found that the BnRALF10 peptide, derived from the C-terminus of BnPRORALF10, could stimulate ROS burst, intracellular Ca^2+^ elevation, MAPK activation and defense-related gene expression induction (Figure 6). The transcriptional level of *BnRALF10* was significantly enhanced by fungal infection (Figure 3), and pretreatment of BnRALF10 could induce resistance to *S. sclerotiorum* in oilseed rape leaves (Figure 5). These effects of BnRALF10 are comparable with AtPep1, the well-characterized DAMP (Bartels and Boller, 2015), indicating that BnRALF10 functions as a DAMP. Notably, the role of RALFs in plant defense reported to date is all negative. For example, AtRALF23 inhibits plant resistance to biotrophic pathogen *Pseudomonas syringae* through its receptor FER in a S1P-dependent manner (Stegmann et al., 2017); It has also been shown to stabilize the basic helix-loop-helix transcription factor MYC2 and promote JA signaling *via* FER, and play a negative role in plant immunity to *P. syringae* (Guo et al., 2018). Silencing of the *Fragaria* × *ananassa* RALF-33-like gene in red fruits of strawberry led to a delay in fruit colonization by fungal pathogen *Colletotrichum acutatum* (Merino et al., 2019). F-RALF from *Fusarium oxysporum* promotes fungal virulence and suppresses plant immune responses through the FER receptor kinase (Masachis et al., 2016); RALF-like peptides secreted by root-knot nematode (RKN) facilitate parasitism through the plant receptor FER (Zhang X. et al., 2020); RALF-like 1 from RKN uses soybean receptor kinase GmLMM1 as susceptible target to promote parasitism in soybean (Zhang X. et al., 2021). Intriguingly, here, we provide evidence that BnRALF10 interacts with BnFER and knock-down of *BnFER* renders BnRALF10 induce less ROS and lower resistance to *S. sclerotiorum*, revealing that BnRALF10, when recognized by BnFER, plays a positive role in resistance to the necrotrophic fungal pathogen *S. sclerotiorum*. Our results demonstrate that RALFs plays distinct roles in plant immunity.

### Mechanisms Underlying BnRALF10-Stimulated Immunity

Although great insights have been unveiled for the maturation and recognition of RALFs (Haruta et al., 2014; Stegmann et al., 2017; Xiao et al., 2019), the signaling downstream the recognition remains largely unknown. To understand how BnRALF10 stimulates plant immunity to *S. sclerotiorum*, we performed quantitative proteomics analysis to detect the BnRALF10-elicited defense (RED) proteins, which significantly change in protein level between oilseed rape leaves pretreated with ddH_2_O and BnRALF10 peptide. Interestingly, among the 314 differentially expressed proteins (DEPs) (Supplementary Table 2), dozens of functionally proved RED proteins were identified and notably some novel potential defense mechanisms underlying BnRALF10-stimulated immunity against *S. sclerotiorum* were revealed (Table 2).

One of our interesting findings is that BnRALF10-stimulated immunity is associated with well positioning anchoring of immune proteins to plasma membrane (PM) via synergy of synaptotagmin 1 (SYT1) and syntaxin 121 (SYP121/PEN1). SYP121/PEN1 encodes a syntaxin localized at PM. As a member of the SNARE superfamily, it forms a complex with the PM-localized SNAP33, and the vesicle-residing VAMP721/722, which constitutes the exocytic secretion system to secrete immune molecules for immunity against a variety of pathogens (Johansson et al., 2014; Yun et al., 2016). SYT1 encodes a membrane trafficking proteins specifically localized to the ER-PM boundary. It regulates endocytosis endosome recycling at PM. The Arabidopsis SYT1 binds PEN1 and fine-tunes the PEN1-SNAP33-VAMP721/722 exocytic activity (Kim et al., 2016; Yun et al., 2016). In this context, it is interesting that BnRALF10 significantly altered the abundance of both SYT1 and SYP121 as well as other SYPs such as SYP21, 22, 61 and v-SNARE 13 (Table 2 and Supplementary Table 2). It is likely that BnRALF10 activates SYT1-SYP121-mediated secretion system to guarantee positioning anchoring and abundance of immune proteins at PM and probably other membranes thereby stimulate immune responses. To date, little is known about the immune molecules secreted by this system. In Arabidopsis, SNAREs SYP61 and SYP121 directly bind to and coordinate the trafficking of PM aquaporin PIP2;7, a water channel, to modulate the cell membrane water permeability (Hachez et al., 2014). Coincidently, BnRALF10 infiltration reduced abundance of the aquaporin PIP2;7 protein (Supplementary Table 2). This implies that BnRALF10 might regulate the water access to the infected pathogen to inhibit pathogen via SYP61-SYP121-mediated suppression of PIP2;7. Furthermore, the SYP121-SNAP33-VAMP721/722 exocytosis module directly interacts and controls PM localization and function of K^+^ channels (Grefen et al., 2015; Zhang et al., 2015). Whether calcium channels or transporters, like K^+^ channels, are regulated by SYT1 and SYP121-SNAP33-VAMP721/722 is a intriguing issue to be addressed, considering the pivotal role of calcium signaling pathways in BnRALF10-elicited immunity (refer to below for details, Table 2 and Supplementary Table 2). Additionally, homeostasis of some PM-localized immune receptors are regulated by PEN1 and other protein secretion systems. For instance, abundance of FLS2, the receptor of flg22, at PM is regulated by PEN1- and EXO70B1/EXO70B2-mediated exocyst pathways (Wang et al., 2020). Under this consideration, whether FER, the likely receptor of BnRALF10, is regulated by PEN1 and other exocyst pathways is worth further study. Moreover, it is also interesting to check whether other PM-localized RED proteins are modulated by the SYT1 and SYP121/PEN1-SNAP33-VAMP721/722 exocytosis systems.

Linked to the above point, another interesting finding is that BnRALF10-stimulated immunity is tightly associated with optimal membrane compartments creating for immune receptor complex formation and immune signal transduction via REMs and PRFs. REMs modulate PM nanodomain organization to promote formin condensation for actin remodeling in innate immune responses (Ma et al., 2022) and to enable the pathogen-derived outer membrane vesicles (OMVs) insert into the host PM, thereby alters host membrane properties to potentiate plant immune responses (Tran et al., 2022). In contrast, PRFs negatively regulate formin-mediated actin assembly (Sun et al., 2018). Intriguingly, BnRALF10 enhanced REMs abundance while reduced PRFs accumulation (Table 2). Together, these results reveal that BnRALF10 fine tunes REM- and PRFs-dependent actin cytoskeleton to promote plant immunity. In addition, REMs were reported to trigger immunity by polymerizing and physically interacting with receptor-like kinases (RLK) (Yu, 2020). Their phospho-status defines their PM nanodomain organization and activities in restricting viral cell-to-cell movement (Perraki et al., 2018) as well as in enhancing RBOHB-dependent ROS production (Cai et al., 2020). In this scenario, it is possible that BnRALF10 recognition somehow causes phosphorylation of REMs, which promotes their functions including creating optimal membrane compartments for immune complex formation and immune signal transduction, thereby induces immunity. Some RALFs modulate flg22-triggered immunity (Stegmann et al., 2017). It is likely that RALF recognition complex RALF-FER-LLGs localizes together with flg22 recognition complex flg22-FLS2-BAK1 in the same nanodomain on PM, which is separated from the nanodomain containing BRI1 complex for growth regulation and even other immunity receptor complexes (Bücherl et al., 2017).

Twelve proteins involved in redox homeostasis are enriched in the collection of proteins differentially expressed in between ddH_2_O and BnRALF10 peptide-treated leaves. These proteins include ROS generator peroxisomal (S)-2-hydroxy-acid oxidase GLO2 and a variety of antioxidants such as peroxidases, superoxide dismutase [Mn] 1, GSTs, thioredoxins and glutaredoxin (Table 2 and Supplementary Table 2). ROS is pivotal in plant-*S. sclerotiorum* interactions (Williams et al., 2011; Wang Z. et al., 2019; Ding et al., 2020). In this regard, it is noticeable that Arabidopsis GLO2 acts as a ROS producer in non-host resistance (Rojas et al., 2012). Intriguingly, its orthologs in *Nicotiana benthamiana*, NbGOX4, plays positive role in resistance to *S. sclerotiorum* (Xu Y. P. et al., 2018). These results suggest that GLO2 likely functions in BnRALF10-elicited ROS and plant immunity to *S. sclerotiorum.* This is an interesting reinforce on understanding the generation mechanisms of pattern-triggered ROS. Additionally, it has been reported that the activities of peroxidase and superoxide dismutase were higher in *S. sclerotiorum*-resistant *B. napus* genotypes compared with *S. sclerotiorum*-susceptible *B. napus* genotypes (Garg et al., 2013). Our previous proteomic analysis for oilseed rape inoculated with *S. sclerotiorum* and its avirulent strain EP-1PB showed that copper-zinc superoxide dismutase, thioredoxin 1 and glutaredoxin 3 were up-regulated in the latter, indicating the important role of antioxidant capacity of *B. napus* in defense against *S. sclerotiorum* (Cao et al., 2016). BnRALF10 elicits ROS burst in oilseed rape leaves (Figure 6). The fact that these ROS scavenging related enzymes are induced by BnRALF10 treatment may indicate their participation in the ROS homeostasis regulation during BnRALF10-stimulated immunity to *S. sclerotiorum*.

Rapid alkalinization factor-triggered immunity has been connected closely with Ca^2+^ signaling pathway. Previous reports have established that RALF peptides induce Ca^2+^ influx across the PM and the release of Ca^2+^ from intracellular reserves (Haruta et al., 2008; Shih et al., 2014). Here our results also verified this conclusion (Figure 6). Gjetting et al. (2020) showed that the rapid burst of intracellular Ca^2+^ preceded apoplastic alkalinization in roots triggered by RALFs, and the inhibition of H^+^-ATPase activity mediated by RALF involves an obligatory Ca^2+^ signal. Additionally, our previous studies reveal that Ca^2+^ signaling is essential to plant defense against *S. sclerotiorum*. Ca^2+^ signaling genes encoding calmodulins (CaMs), Ca^2+^-dependent protein kinase (CDPK)-related kinases (CRKs), calcium and calmodulin-dependent protein kinases (CCaMKs), cyclic nucleotide-gated channels (CNGCs), and calmodulin-binding transcription activator 3 (CAMTA3) significantly affect plant resistance to *S. sclerotiorum* (Zhao et al., 2013; Saand et al., 2015a,b; Wang et al., 2015, 2016; Rahman et al., 2016). Our current proteomics analysis demonstrates that annexin D1 (Ca^2+^-dependent phospholipid-binding protein) and synaptotagmin-1 (Ca^2+^ sensors), two important components of Ca^2+^ signaling pathway, were induced in *B. napus* infiltrated with BnRALF10 (Supplementary Table 4). Synaptotagmin-1 and annexins have been found to play a negative role in plant immunity to biotrophic pathogens (Levy et al., 2015; Kim et al., 2016; Zhao et al., 2021), while its role in plant defense against necrotrophic pathogens remains unknown. Therefore, it will be intriguing to further confirm the role of synaptotagmin-1 and annexin D1 in *B. napus* defense against *S. sclerotiorum* and dissect their functional mechanisms, especially in BnRALF10 evoked Ca^2+^ signaling.

Based on our results, it is proposed that BnRALF10 alters abundance of RED proteins, which ensures anchoring and abundance of immune proteins at PM via synergy of SYT1 and SYP121/PEN1, optimizes membrane compartments for immune receptor complex formation and immune signal transduction via REMs and PRFs, elicits various immune responses including ROS burst and cytosolic Ca^2+^ promotion, thereby stimulate plant immunity to pathogens including *S. sclerotiorum*.

## Conclusion

The oilseed rape genome harbored 61 RALFs. Half of them (belonging to clades III and IV) were atypical, containing a less conserved YISY motif and lacking a RRXL motif or a pair of cysteines. Expression profiles of RALF family in response to pathogen and molecular patterns were RALF- and stimulus-dependent. In general, *BnRALF* genes were expressionally up-regulated by *S. sclerotiorum*, while down-regulated by BnPep5 and SsNLP1. Thus, BnRALF members are likely differentially involved in plant immunity. The canonical RALF, BnRALF10, induced diverse immune responses such as ROS accumulation, cytosolic Ca^2+^ promotion, MAPK activation, defense-related gene expression induction and stimulated resistance to *S. sclerotiorum*, and thus likely functioned as a DAMP to play a positive role in plant immunity. Moreover, BnFER was likely a receptor of BnRALF10. Quantitative proteomic analysis identified dozens of BnRALF10-elicited defense (RED) proteins. BnRALF10 modulated RED protein abundance to fine-tune plant immunity.

## Data Availability Statement

The original contributions presented in the study are included in the article/Supplementary Material, further inquiries can be directed to the corresponding author/s.

## Author Contributions

X-ZC coordinated the project. Y-HH conducted the bioinformatics, gene expression, and immune response analyses. Y-HH and Z-RZ performed disease resistance evaluation. Y-HH, S-YC, and Y-PX conducted the proteomic analysis. X-ZC conceived of the study and participated in its design and coordination. X-ZC and Y-HH prepared the manuscript. All authors read and approved the final manuscript.

## Conflict of Interest

The authors declare that the research was conducted in the absence of any commercial or financial relationships that could be construed as a potential conflict of interest.

## Publisher’s Note

All claims expressed in this article are solely those of the authors and do not necessarily represent those of their affiliated organizations, or those of the publisher, the editors and the reviewers. Any product that may be evaluated in this article, or claim that may be made by its manufacturer, is not guaranteed or endorsed by the publisher.

## Footnotes

1
http://www.arabidopsis.org/

2
https://www.ncbi.nlm.nih.gov/

3
http://au.expasy.org/tools/pi_tool.html

4
https://itol.embl.de/

5
http://nrbsc.org/gfx/genedoc

6
http://weblogo.threeplusone.com/

7
https://imagej.net
