# Supplementary material for: Genome-Wide Identification of Rapid Alkalinization Factor Family in Brassica napus and Functional Analysis of BnRALF10 in Immunity to Sclerotinia sclerotiorum
Source: Front Plant Sci. 2022 May 3;13:877404. doi: 10.3389/fpls.2022.877404 (PMC9113046; doi:10.3389/fpls.2022.877404)
Supplement: Supplementary file 6 [file Image_1.pdf]

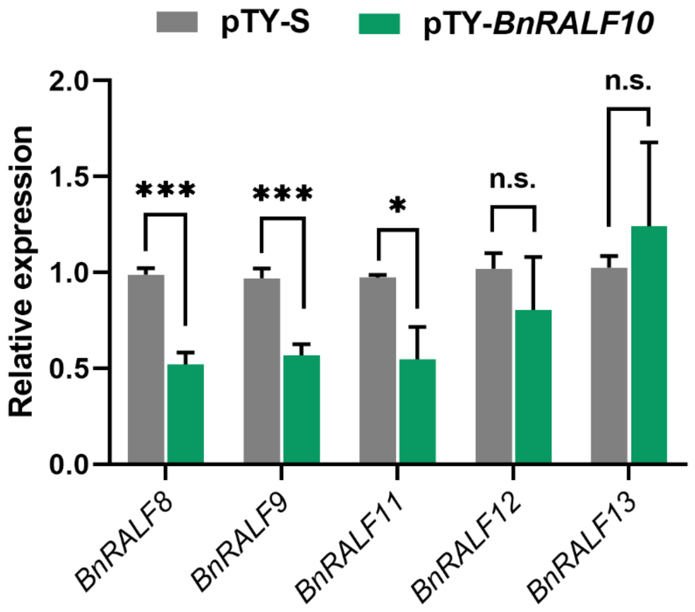

Figure S1. Expression of *BnRALF10* homologous genes in rape plants VIGS treated with pTY-*BnRALF10*. Data are statistically analyzed by Student's *t*-test (n = 3) and shown as the mean ± SE. The asterisks indicate significant differences (*$P \leq 0.05$, ***$P \leq 0.001$). n.s., not significant.

A

	Description	Scientific Name	Max Score	Total Score	Query Cover	E value	Per. Ident	Acc. Len	Accession
☑	hypothetical protein HID58_081986 [Brassica napus]	Brassica napus	1582	1582	100%	0.0	88.50%	1893	KAH0864775.1
☑	unnamed protein product [Brassica napus]	Brassica napus	1572	1572	96%	0.0	88.82%	1994	CAF2046803.1
☑	unnamed protein product [Brassica napus]	Brassica napus	1569	1569	96%	0.0	88.71%	1207	CAF2111057.1
☑	unnamed protein product [Brassica napus]	Brassica napus	1568	1568	96%	0.0	89.30%	893	CAF2151284.1
☑	receptor-like protein kinase FERONIA isoform X2 [Brassica napus]	Brassica napus	1567	1567	96%	0.0	89.30%	892	XP_013734398.1

B

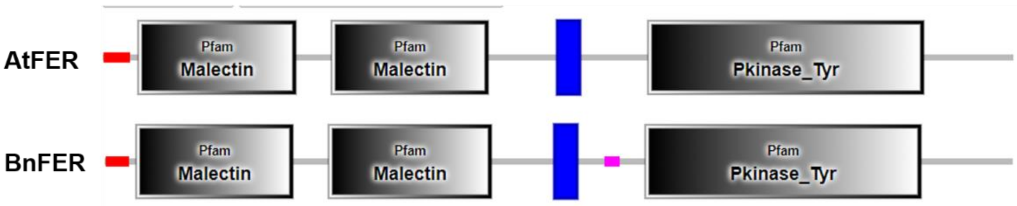

Figure S2. *B. napus* and *A. thaliana* FERs. (A) Result of BLASTp search for *B. napus* FER using *A. thaliana* FER as query. Red-boxed (A) is the FER in *B. napus* cultivar Zhongshuang 11. (B) Domain composition of *B. napus* and *A. thaliana* FERs.

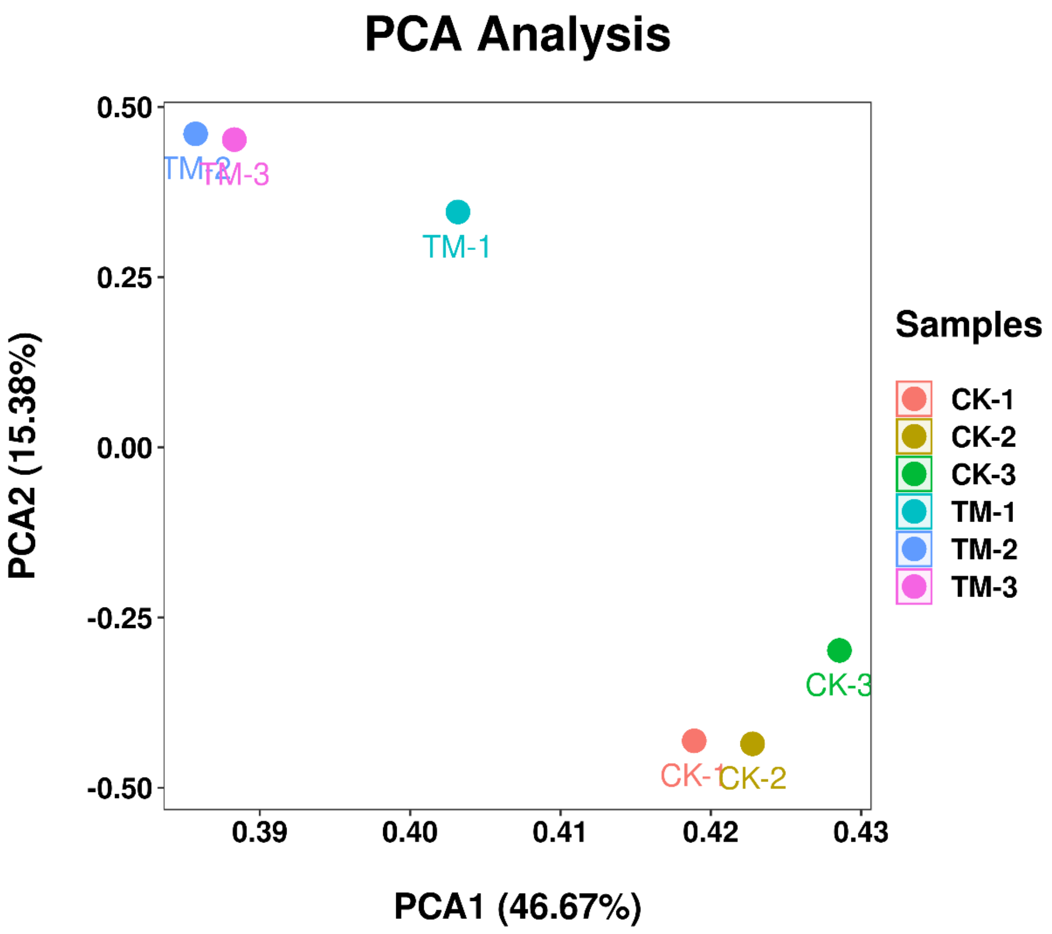

Figure S3. Results of Principal Components Analysis of samples for quantitative proteome analysis. TM: BnRALF10-treated leaves; CK: ddH_2O-treated leaves.

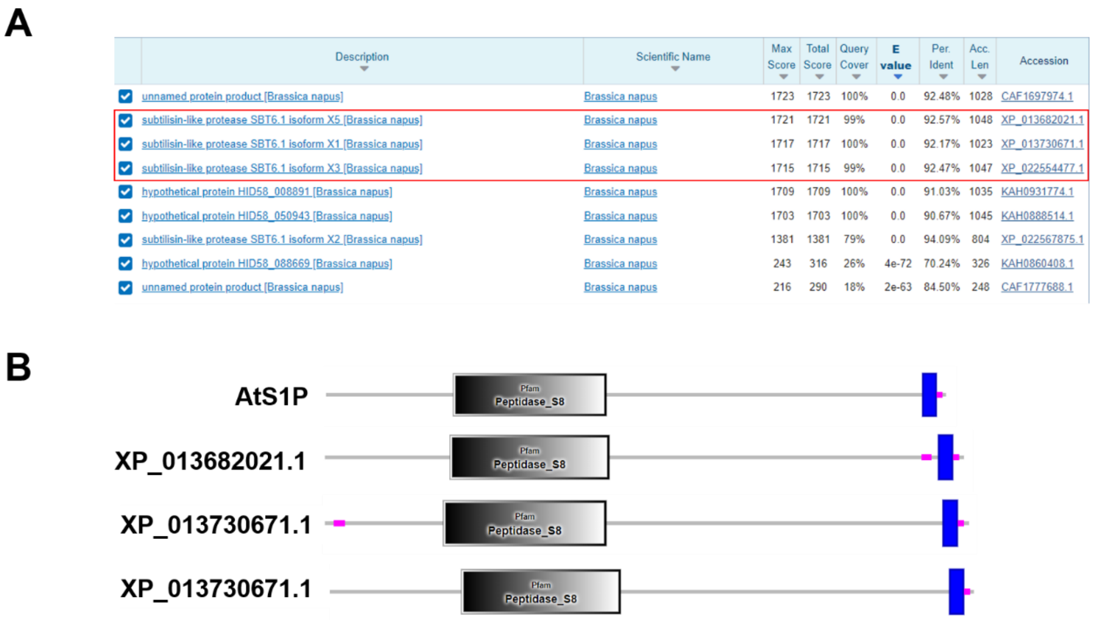

A

	Description	Scientific Name	Max Score	Total Score	Query Cover	E value	Per. Ident	Acc. Len	Accession
☑	unnamed protein product [Brassica napus]	Brassica napus	1723	1723	100%	0.0	92.48%	1028	CAF1697974.1
☑	subtilisin-like protease SBT6.1 isoform X5 [Brassica napus]	Brassica napus	1721	1721	99%	0.0	92.57%	1048	XP_013682021.1
☑	subtilisin-like protease SBT6.1 isoform X1 [Brassica napus]	Brassica napus	1717	1717	100%	0.0	92.17%	1023	XP_013730671.1
☑	subtilisin-like protease SBT6.1 isoform X3 [Brassica napus]	Brassica napus	1715	1715	99%	0.0	92.47%	1047	XP_022554477.1
☑	hypothetical protein HID58_008891 [Brassica napus]	Brassica napus	1709	1709	100%	0.0	91.03%	1035	KAH0931774.1
☑	hypothetical protein HID58_050943 [Brassica napus]	Brassica napus	1703	1703	100%	0.0	90.67%	1045	KAH0888514.1
☑	subtilisin-like protease SBT6.1 isoform X2 [Brassica napus]	Brassica napus	1381	1381	79%	0.0	94.09%	804	XP_022567875.1
☑	hypothetical protein HID58_088669 [Brassica napus]	Brassica napus	243	316	26%	4e-72	70.24%	326	KAH0860408.1
☑	unnamed protein product [Brassica napus]	Brassica napus	216	290	18%	2e-63	84.50%	248	CAF1777688.1

Figure S4. ***B. napus* and *A. thaliana* S1Ps.** (A) Result of BLASTp search for *B. napus* S1Ps using *A. thaliana* S1P as query. Red-boxed (A) is the S1Ps in *B. napus* cultivar Zhongshuang 11. (B) Domain composition of *B. napus* and *A. thaliana* S1Ps.
